# Somatic Embryogenesis as Key Technology for Shaping the Rubber Tree of the Future

**DOI:** 10.3389/fpls.2018.01804

**Published:** 2018-12-04

**Authors:** Eline Mignon, Stefaan Werbrouck

**Affiliations:** ^1^Socfinco, Fribourg, Switzerland; ^2^Laboratory for Applied In Vitro Plant Biotechnology, Department of Bioscience Engineering, Ghent University, Ghent, Belgium

**Keywords:** *Hevea brasiliensis*, rejuvenation, micropropagation, yield, rubber

## Abstract

Worldwide, *Hevea* producers face the need to replant large surfaces in the coming years. The rubber yield per ha, produced by trees grafted on heterogeneous illegitimate seedling rootstocks, has reached its maximum. For long-standing Hevea clones, as for a lot of other tree species, one of the consequences of physiological aging is reduced *in vitro* growth and the lack of a proper geotropic (tap) root system. Somatic embryogenesis on young inner seed integument or stamen filaments provides a mean to regain ontogenetic juvenility. The process is limited by irregular germination of the somatic embryos. Nevertheless, with the obtained *in vitro* plants, juvenile lines have been established of the most important profitable rubber tree clones. Currently they are micropropagated on a commercial scale. Moreover, the produced plants can serve as mother plants for propagation by means of macro-cutting. Somatic embryogenesis enables the production of transgenic *Hevea brasiliensis* as well. Genes conferring plant disease resistance, abiotic stress tolerance and production of foreign proteins in the lactiferous vessels will further shape the rubber tree of the future.

## Introduction

*Hevea brasiliensis* is the major source of latex, which is still the paramount raw material for more than 40,000 products (Mooibroek and Cornish, 2000). Primarily due to its molecular structure and high molecular weight (>1 million daltons) it has elasticity, abrasion resistance, and impact resistance that cannot easily be surpassed by artificial polymers. Rubber tree plantations cover immense areas in South East Asia and West Africa. Worldwide, rubber producers face the need to replant large surfaces in the coming years. The rubber yield per ha, produced by trees grafted on heterogeneous illegitimate seedling rootstocks, has reached its maximum and the time is right to make a major yield leap. As will be described hereafter, somatic embryogenesis will play an important role in this evolution.

The first rubber plantations were set up in southeast Asia between 1890 and 1930, from seeds of uncontrolled origin. Very early, the need was recognized to propagate rubber trees vegetatively, in order to exploit superior genotypes. For more than fifty years, numerous attempts have been made worldwide to root cuttings from selected mature trees. However, rooting rates were low and a tap root was missing (Muzik and Cruzado, 1958; Seneviratne, 1996). As for most trees, this is linked to the juvenile/mature gradient from trunk to crown (Haffner et al., 1991). As a consequence, the produced trees were highly susceptible to drought and uprooting by tropical storms. Such rooting problems did not occur when cuttings were taken from juvenile seedlings, which were though useless because of their low average inherent quality. This is caused by the heterozygous parents. Up to recent times, Hevea clones are propagated by grafting on seedling rootstocks (Priyadarshan, 2017). The inherent heterogeneity of the rootstock is the main cause of intraclonal variability regarding growth vigor and yield. In an experiment with ‘RRII105,’ total volume of latex and dry rubber harvest ranged between 5.0 to 325.0 ml and 1.8 to 144.0 g, respectively (Chandrashekar et al., 1997). Besides, also a reduction of latex yield when the tree was tapped was observed at the level of the graft union.

When *in vitro* research started in Hevea, the same problems appeared as with the cuttings. Quite a few reports are available on *in vitro* propagation of seedlings. As can be expected, seedlings could generally be multiplied *in vitro*, but shoots that were initiated from mature elite clones were very much recalcitrant. The few produced plants failed to produce gravitropic roots (Nayanakantha and Seneviratne, 2009). Then, the insight arose that the mature elite varieties had to be rejuvenated. Somatic embryogenesis is the most efficient method for rejuvenation as the derived plants can be considered as ontogenetically juvenile (Lardet et al., 2009; Monteuuis et al., 2011).

## Somatic Embryogenesis

### Introduction

An efficient plant regeneration pathway through somatic embryogenesis is essential for rejuvenation. In an easy tree species, such as *Coffea* (Van Boxtel and Berthouly, 1996), young leaves can be used to induce embryogenic callus. However, in most tree species, once the phase change to maturity has been passed, there is a decline in the potential of leaf and shoot explants for somatic embryogenesis. Exceptions are tissues that are closely associated with the reproductive organs. In *Castanea sativa* these are stamen filaments (Jörgensen, 1989), in *Theobroma cacao* staminodes are isolated (Li et al., 1998). In *Quercus ilex* young integuments are reactive (Barra-Jiménez et al., 2014) and in *Rosa hybrida* petals are used (Murali et al., 1996). In Hevea, up to now, only immature seed integuments and stamen filaments proved to be responsive.

There are two pathways to obtain primary somatic embryos. In the case of direct somatic embryogenesis, primary somatic embryos appear in limited numbers directly on the explant, after a limited callus phase. This intermediate callus interphase should be restricted, in order to minimize the risk of somaclonal variation (Carron et al., 2009). In case of ‘indirect embryogenesis,’ somatic embryos develop on selected and subcultured ‘embryogenic’ callus lines that were induced on the explants. This technique allows cryopreservation (Lardet et al., 2007) and genetic transformation (Montoro et al., 2003), but repeated callus culture increases the risk for mutations and epigenetic changes (Carron et al., 2000). Although of less practical importance, secondary and tertiary somatic embryogenesis can be generated when somatic embryo fragments are used as explants. In this way, an average of 10 new somatic embryos was achieved per cultured somatic embryo (Hua et al., 2010).

### Direct Primary Somatic Embryogenesis

#### Immature Seed Integument

**FIGURE 1:**
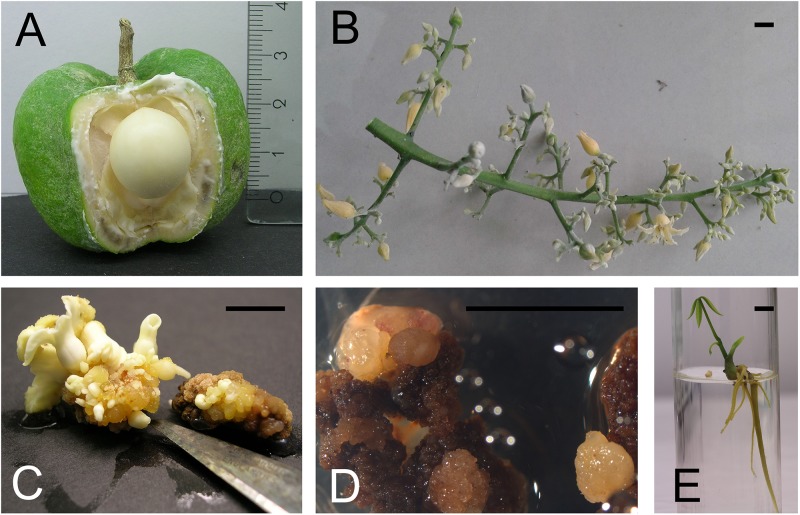
Somatic embryogenesis in *Hevea brasiliensis*. **(A)** Immature rubber tree fruit and seed. **(B)** The inflorescence includes distinct male and female flowers. **(C)** Different stages of somatic embryos appear on the secondary integument. **(D)** Somatic embryogenesis starts on anther filaments. **(E)** A germinated somatic embryo. (pictures E. Mignon). Bar = 5 mm.

The fruit is a capsule that usually contains three carpels each inclosing a seed (Muzik, 1954). They are harvested before the inner fruit wall starts lignifying. At that time, there is still a lot of space in the cavity of the carpel (Figure 1A). After surface sterilization by rinsing in ethanol 70%, incubating for 15 min in 10% commercial bleach (8°) and rinsing, the immature fruits are opened. The white seeds, which should have a diameter of about 1 cm are sterilized in the same way as the fruits. The nearly indistinguishable small embryo is laying in a semi-liquid to gelatinous endosperm lining the central cavity of the seed. It is removed by cutting away the basal part of the seed, at the funiculus side. Thin slices of the remaining inner part of seed integuments are taken as explant. The five subsequent media as defined by Carron et al. (1995) are a good starting point to optimize the protocol for direct somatic embryogenesis, as it is only reliable for a few genotypes. They consist of an embryogenic callus induction phase, remarkably on a medium with 3,4-D instead of the usual 2,4-D. Also during the somatic embryo expression phase (Figure 1C) and the ‘pro-embryo development phase,’ 3,4-D stays present in this protocol, albeit in reduced concentrations. The maturation and germination phase (Figure 1E) complete the process which can easily take one year. Carron et al. (1995) mentioned that of clones ‘RRIM600’ and ‘PB260,’ respectively 7, 6, and 14,9% of the explants formed shoots. Dibi et al. (2010) compared self-rooted *in vitro* plantlets originating from integument derived somatic embryos with conventional mature budded clones. The trunk of the *in vitro* plants gained 9.93–16.83% and a gain of dry rubber production per tree of 3.5–32.35% was recorded.

#### Stamen Filament

The inflorescence includes distinct male and female flowers. Both are creamy-yellow and lack petals (Figure 1B). They are so small that it requires a binocular microscope to isolate the stamens. The main disadvantages of using inflorescences is the very limited time frame of availability. Jayasree et al. (1999) obtained high frequency somatic embryogenesis (24% of the explants responded) and plant regeneration from immature stamens (Figure 1D). Callus was induced on a modified Murashige and Skoog (1962) (MS) medium with 2,4-D and Kin. Optimal embryo induction was achieved on medium supplemented with NAA and Kin. Further development of the embryos into plantlets was achieved on a hormone-free medium and plants could be established in the field. Cytological analysis revealed that all the plantlets tested were diploid. In principle, fingerprinting should also be added to ploidy analysis, because immature pollen could give rise to dihaploid callus and ditto embryos (Chen, 1990). Already in 2001, Xiongting et al. (2001) assessed the field performance of rubber trees derived from stamen derived somatic embryos. Self-rooted juvenile clones were compared with their grafted donor clones. The stem girth of the self-rooting juvenile clones at 50 cm above ground was 109.1–135.2% of that of the donor clones. The self-rooting juvenile clones produced an average dry rubber yield per tree per year 129.9–146.3% of the donor clone in the first 4 years.

### Indirect Primary Somatic Embryogenesis

#### Embryogenic Callus Lines

Montoro et al. (1993) developed an indirect somatic embryogenesis protocol in which seed integuments derived explants were used to produce friable embryogenic calli, that could be maintained by regular subcultures. The team demonstrated that a high calcium concentration is essential to obtain friable callus with embryogenic potential and reported about the beneficial effects of maltose (Blanc et al., 2002). Etienne et al. (1997) optimized temporary immersion systems for callus proliferation and somatic embryo development. To avoid loss of regeneration competence during long-term maintenance of these friable embryogenic callus lines, Lardet et al. (2007) developed a cryopreservation method. They showed that a pre-treatment on al low calcium medium (1 mM) or even without calcium is essential for post-thaw recovery of friable callus. Probably a concomitant reduction in endogenous calcium lead to a greater cryotolerance and an increased post-thaw embryogenic competence and plant regeneration. Despite many efforts, this pathway is still limited to a few clones and the observed regeneration rates are highly variable. From the most reactive clone PB 260, almost 17,000 whole plants were produced and compared with budded plants in more than 15 ha field trials (Carron et al., 2009). This revealed apparent somaclonal variation regarding branch architecture, shape and color of the leaves, growth vigor and root quality. So far this experience has prevented mass production via this pathway.

#### Protoplast Regeneration

Sushamakumari et al. (2000) showed for the first time how to regenerate plants from protoplast of *H. brasiliensis*. Embryogenic calli were induced on immature inflorescences and inner integuments of immature seeds. The callus cell walls were enzymatically digested and the resulting protoplasts were cultured on a nitrocellulose membranes overlying a semi-solid medium containing *Lolium multiflorum* nurse cells. 40% of the derived calli developed somatic embryos upon transfer to MS-based regeneration medium. After 3 months of culture germinated plants were obtained.

### Secondary Somatic Embryogenesis

Secondary somatic embryogenesis allows to produce an unlimited number of secondary somatic embryos in a cyclic routine as was demonstrated by Hua et al. (2010). Stamen derived cotyledonary somatic embryos were cut into 3.0 × 3.0-mm fragments and used for induction of secondary embryogenesis. This was repeated with the obtained secondary somatic embryos. Although each embryo fragment produced no more then 0,67 new mature cotyledonary embryos, the general multiplication factor was higher than 10 in each cycle. At present, the low efficiency of plantlet recovery from somatic embryos still remains a limits for large scale industrial application of this pathway.

## Microcutting and Rooting

Although microcutting is an easy procedure for many plant species, rubber trees are rather recalcitrant. In principle, one rejuvenated mother plant per clone is enough to start the microcutting process, which leads to a logarithmically increasing number of clones.

Generally, temporary immersion bioreactors can improve the multiplication rates of a number of herbaceous crops in a spectacular way. The principle reason is the combination of an improved nutrient supply with forced aeration of the headspace. Also for woody plants such as *Hevea*, this technology promised a breakthrough. Nevertheless, a standard solid medium multiplication protocol cannot simply be copy/pasted to a liquid medium system. Problems to be solved are small shoots, hyperhydricity, excessive leaf drop and break out of endogenous bacterial contamination (Máximo et al., 2018).

By comparing the root architecture of seedlings and somatic embryo derived *in vitro* plants in the field, (Carron et al., 2000) showed that they are similar in producing a tap root. This is a typical juvenile feature and suggest that the passage through somatic embryogenesis is truly rejuvenating. However, the assessment of the field performance of *in vitro* plants revealed that the tap root might be damaged by careless planting practices. During all post vitro transfer steps, it’s critical to give special attention to the root, not only *in vitro*, but also during acclimatization, hardening and field planting.

Certification of the mother trees and a strict harvest protocol are essential to reduce causes of variations. Factors to take into account during harvest are mother tree age (Lardet et al., 2009), fruit stage, fruit freshness and transport conditions. Nonetheless, ‘fruit within tree’ and ‘year’ (climate) effects are still a concern, because there is still a rather large variation in somatic embryo yield within treatments and clones. Fingerprinting of the germinated somatic embryos before starting mass cloning is advisable, to exclude labeling and other human errors during immature fruit or inflorescence harvest and laboratory manipulations (Besse et al., 1993; Roy et al., 2004; Li et al., 2014).

## Budwood and Cutting Gardens

In order to exploit the benefits of rejuvenated plants, Masson et al. (2013) established gardens with rejuvenated mother plants to provide cuttings. After 3 weeks under suitable rooting conditions in nursery, rooting rates of 75% were obtained for two established clones. The cuttings produced vigorous and taproot-like adventitious roots and continued to grow vigorously. It was projected that, to produce cuttings to plant 10 ha, 150 m2 is required for mother plants, and 50 m2 for nursery vs. 1400m2 of budwood garden and 800 m2 for the production of grafted plants. As the multiplication rate by cuttings is low, limited by strong apical dominance that controls the number of stems per mother plant (Montoro et al., 2012), this approach requires a considerable surface to grow mother plants. If this problem could be solved, it would allow to reduce the cost price of a tree. Likewise, for coffee the strategy to produce horticultural rooted mini-cuttings from somatic embryo derived mother plants was proposed (Georget et al., 2017).

## Genetic Transformation and Genome Editing

Conventional breeding in Hevea is rather difficult due to the heterozygous nature of Hevea and its long juvenile phase. Since indirect somatic embryogenesis was discovered, the potential for genetic transformation was realized. Agrobacterium-mediated gene transfer has been the best method for delivering foreign genes into Hevea. Hevea was transformed with marker genes just as GUS and GFP but since then the research focused on transferring important agronomic genes such as enhanced tolerance to abiotic stress, high latex and timber yield and even the production of recombinant proteins. For reviews we refer to Venkatachalam et al. (2007) and Montoro et al. (2012). The clone ‘PB260,’ which is very suitable to produce friable callus lines with a high plant regeneration capacity, is ideal for transformation. Currently, friable callus lines are precultured for 15 days on a CaCl2-free medium with BA and 3,4-D. Then, small cell aggregates are cocultured for 5 days with *Agrobacterium tumefaciens* and transgenic lines are established after a number of subcultures of 3 weeks on a selective medium. Somatic embryos are induced on these callus lines and after germination, genetic modified plants are obtained (Lestari et al., 2018; Martin et al., 2018). As we are now entering the age of genome editing, protoplast regeneration could offer a tool to modify DNA in *Hevea* without entering foreign DNA. This may alleviate the regulatory concerns related to genetically modified plants which are now seriously retarding the application of genetic modified *Hevea*.

## Conclusion and Future Prospects

On the whole, the intensive efforts invested in studying somatic embryogenesis turned out to be very interesting for the *Hevea* industry. The associated rejuvenation allows vegetative multiplication of elite trees, cryopreservation, genetic modification, and genome editing. Although the fundaments of somatic embryogenesis mediated rejuvenation of *H. brasiliensis* were established 35 years ago, industrial clones have only been systematic rejuvenated during the last decade. The process of *Hevea* cloning is time consuming: somatic embryos are regenerated from immature seed integuments, slowly germinated somatic embryos have to be micropropagated, *in vitro* shoots have to be rooted and are planted after acclimatization to grow out into plantation trees. Moreover, the published protocols are based on trial and error using a limited number of cultivars. This genotype dependence implies that for every new genotype, often painstaking optimization of the basic protocol has to be performed. A number of reports have confirmed that elite clones grow faster and yield more latex when growing on own roots than when budded on seedlings. (Xiongting et al., 2001; Dibi et al., 2010; Montoro et al., 2012). This stimulated the establishment of large scale comparative field trials in Ivory Coast. Data will soon be available to compare differences in latex yield between grafted and micropropagated trees. This will help to decide how to redirect worldwide Hevea production, all thanks to somatic embryogenesis.

## Author Contributions

SW and EM wrote the manuscript. EM delivered the photos.

## Conflict of Interest Statement

The authors declare that the research was conducted in the absence of any commercial or financial relationships that could be construed as a potential conflict of interest.
